# Cancer treatment data available in European cancer registries: Where are we and where are we going?

**DOI:** 10.3389/fonc.2023.1109978

**Published:** 2023-02-08

**Authors:** Francesco Giusti, Carmen Martos, Annalisa Trama, Manola Bettio, Arantza Sanvisens, Riccardo Audisio, Volker Arndt, Silvia Francisci, Carine Dochez, Josepa Ribes, Laura Pareja Fernández, Anna Gavin, Gemma Gatta, Rafael Marcos-Gragera, Yolande Lievens, Claudia Allemani, Roberta De Angelis, Otto Visser, Liesbet Van Eycken, the ENCR Working Group on Treatment Data Harmonisation

**Affiliations:** ^1^ European Commission, Joint Research Centre (JRC), Ispra, Italy; ^2^ Belgian Cancer Registry, Brussels, Belgium; ^3^ Foundation for the Promotion of Health and Biomedical Research in the Valencian Region (FISABIO), Valencia, Spain; ^4^ Evaluative Epidemiology Unit, Department of Research, Fondazione IRCCS Istituto Nazionale dei Tumori, Milan, Italy; ^5^ Epidemiology Unit and Girona Cancer Registry, Catalan Institute of Oncology, Oncology Coordination Plan, Department of Health, Autonomous Government of Catalonia; Girona Biomedical Research Institute (IDIBGI), Girona, Spain; ^6^ Department of Surgery, Institute of Clinical Sciences, Sahlgrenska University Hospital, Göteborg, Sweden; ^7^ Epidemiological Cancer Registry Baden-Württemberg (M110) & Unit of Cancer Survivorship (C071), Division of Clinical Epidemiology and Aging Research (C070), German Cancer Research Center (DKFZ), Heidelberg, Germany; ^8^ National Centre for Disease Prevention and Health Promotion, Istituto Superiore di Sanità, Rome, Italy; ^9^ Catalan Cancer Plan, Department of Health of Catalonia, Hospitalet del Llobregat, Barcelona, Spain; ^10^ Northern Ireland Cancer Registry, Centre for Public Health, Queen’s University Belfast, Belfast, Ireland; ^11^ Department of Radiation Oncology, Ghent University Hospital and Ghent University, Ghent, Belgium; ^12^ Cancer Survival Group, London School of Hygiene and Tropical Medicine, London, United Kingdom; ^13^ Department of Oncology and Molecular Medicine, Istituto Superiore di Sanità, Rome, Italy; ^14^ Department of Registration, Netherlands Comprehensive Cancer Organisation (IKNL), Utrecht, Netherlands

**Keywords:** cancer registry, data harmonisation, questionnaire, big data, Europe, cancer registry data, cancer treament

## Abstract

Population-based cancer registries are responsible for collecting incidence and survival data on all reportable neoplasms within a defined geographical area. During the last decades, the role of cancer registries has evolved beyond monitoring epidemiological indicators, as they are expanding their activities to studies on cancer aetiology, prevention, and quality of care. This expansion relies also on the collection of additional clinical data, such as stage at diagnosis and cancer treatment. While the collection of data on stage, according to international reference classification, is consolidated almost everywhere, data collection on treatment is still very heterogeneous in Europe. This article combines data from a literature review and conference proceedings together with data from 125 European cancer registries contributing to the 2015 ENCR-JRC data call to provide an overview of the status of using and reporting treatment data in population-based cancer registries. The literature review shows that there is an increase in published data on cancer treatment by population-based cancer registries over the years. In addition, the review indicates that treatment data are most often collected for breast cancer, the most frequent cancer in women in Europe, followed by colorectal, prostate and lung cancers, which are also more common. Treatment data are increasingly being reported by cancer registries, though further improvements are required to ensure their complete and harmonised collection. Sufficient financial and human resources are needed to collect and analyse treatment data. Clear registration guidelines are to be made available to increase the availability of real-world treatment data in a harmonised way across Europe.

## 1 Introduction

Among non-communicable diseases, cancer remains one of the most important causes of death worldwide. In 2020, 4 million new cases were estimated to be reported in Europe, with around 1.9 million deaths (1). Although improvements in cancer survival over time are being observed, wide variations between European countries still persist (2–4).

Population-based cancer registries (CRs) are responsible for collecting high-quality population-based incidence and survival data on all reportable neoplasms within a defined catchment-area. Starting from the 1940s, population-based CRs have been operational in an increasing number of European countries, adhering to international standards set by the International Association of Cancer Registries (IACR), in collaboration with the International Agency for Research on Cancer (IARC) (5–8).

Following the European Commission’s 1985 “Europe Against Cancer” Programme, the European Network of Cancer Registries (ENCR) has been operating since 1990 to strengthen the collaboration among CRs, aiming to improve the quality, comparability and availability of cancer incidence data; to provide information on and to monitor cancer incidence and mortality in Europe; and to encourage the use of CRs data in cancer control, health-care planning and research. Since 2012, the ENCR Secretariat has been hosted in Ispra, Italy, by the Directorate-General Joint Research Centre (JRC), the science and knowledge centre of the European Commission. The JRC supports the ENCR with the dissemination and harmonisation of cancer data, with the overall aim of accurately comparing data between European countries. CRs can be members of the ENCR if they are based in countries within the United Nations geographical definition of Europe, plus Cyprus. Currently, nearly 200 population-based CRs are active in Europe, of which 189 are full members and 4 are associate members of ENCR (9, 10). Finally, the JRC has been developing, maintaining and expanding the European Cancer Information System (ECIS) as the infrastructure hosting, processing and disseminating European CR data (1). Harmonised cancer burden indicators across European areas computed from CR data are released in the ECIS web application (11).

During the last decades, the role of CRs has evolved beyond providing cancer incidence and survival data. Depending on available resources, CRs are now becoming more involved in different areas of cancer control, including aetiology of cancer, evaluation of screening programmes, and monitoring quality and outcomes of cancer care and trends in cancer survival (12). In Europe, data collection on cancer treatment modalities (surgery, radiotherapy, chemotherapy, etc…) is very heterogeneous. Several CRs are collecting cancer treatment related data on a continuous or regular basis, while other CRs collect them on an *ad hoc* basis or only for specific projects. Some CRs collect treatment data for all tumours, others only for specific tumours. Data can be collected from medical records and administrative medical claims (such as hospital discharge records and drug prescriptions) (13).

Treatment data collected by CRs allows for the: (1) Monitoring of treatment patterns; (2) Assessment of the compliance with clinical practice guidelines; (3) Evaluation of the impact of new treatments at population level; and (4) Evaluation of access to treatment. Recommended treatment for a specific cancer strongly depends on its stage at diagnosis, as specific treatment modalities and strategies are indicated for selected stages only. The availability of data on stage is therefore a prerequisite for the use and proper interpretation of treatment data collected by CRs (13–15).

Only two previous projects (EUROCHIP-3 and EUROCOURSE) provided an overview on the availability of three main indicators in European population-based CRs: stage at diagnosis, cancer treatment delay and compliance with cancer guidelines. While overall treatment data collection was rather low (30% of CRs), an increase in data collection has been observed (43% of CRs) over time between the two projects (4, 5, 16).

In addition to stage, biomarkers have been playing an important role in guiding treatment options and in the prognosis of several tumour types such as breast, oropharyngeal and lung cancer. Although a constant increase in the number of publications on biomarkers from CRs has been observed in recent years, there is still the need of an harmonisation of such data, and possibly an increased interaction with clinicians and hospital-based registries (17, 18)

Since information about availability and comparability of treatment data is lacking, this article aims to give an overview of the current registration status for cancer treatment data among population-based CRs in Europe. The outcome of the study represents a basis for drafting recommendations to CRs either to initiate data treatment collection or to continue and improve treatment data collection, coding and reporting to assure data comparability among European CRs.

## 2 Methods

To explore the current situation of cancer treatment registration in Europe, a literature search was conducted, including both peer-reviewed articles and mainly cancer registration-related conference proceedings. In addition, treatment data collected in the framework of the 2015 ENCR-JRC data call were explored, and are here summarised.

### 2.1 Literature review

#### 2.1.1 Peer-reviewed literature

A literature review was performed on Pubmed to identify peer-reviewed publications mentioning treatment data from CRs in the title and/or abstract. The first selection was done with keywords “*cancer registry*”, “*cancer registries*”, “*tumor registry*”, “*tumor registries*”, “*tumour registry*”, “*tumour registries*”, “*oncological registry*”, “*oncology registry*”, together with “*treatment*”, “*surgery*”, “*radiotherapy*”, “*chemotherapy*”, “*therapy*”. Keywords with the English language names of all European countries were applied. There was no specific starting period selected, while the end period was set at October 2, 2022.

The results of the Pubmed search were imported in the Rayyan literature review web-tool for further screening (19). As a final step, the results were imported in the statistical software SAS Version 9.3 (SAS Institute Inc., Cary, NC, USA) in order to perform string searches through the *PRXMATCH* Function. A specific search string was also used to look at age groups reported in the publications.

Articles were excluded when the registry was located outside Europe, was not population-based, did not include treatment data, and when the study was not about CR data (e.g. clinical trials).

A consistency check of the selection criteria was independently performed on a sample of 100 articles on which agreement was reached on 97 out of 100 articles. After discussion on the remaining 3 articles, the resulting criteria were applied to the search algorithm.

**Figure 1:**
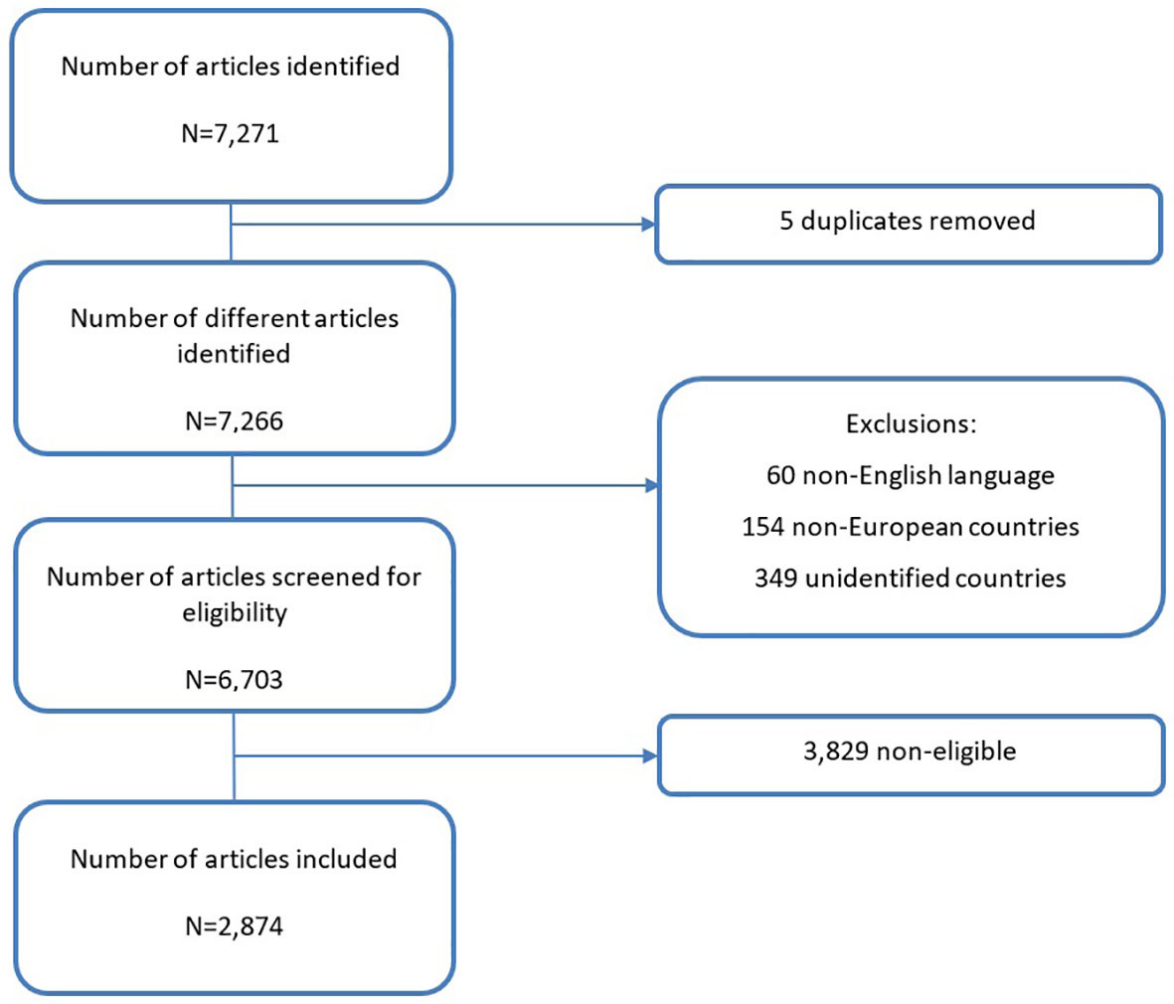
Flowchart of the identification, screening and eligibility of articles included in the literature review.

Articles from population-based CRs in European countries (plus Cyprus) reporting on treatment data were included in the analysis. Articles from CRs operating in more than one country were also included. **Figure 1** describes the flowchart of the included articles.

#### 2.1.2 Conference proceedings

The title and the abstract of presentations given during the period 2016-2019 in scientific international CRs conferences [European Network of Cancer Registries (ENCR), Group for Cancer Epidemiology and Registration in Latin Language Countries (GRELL), the International Association of Cancer Registries (IACR)] plus the European Society for Medical Oncology (ESMO) were also screened.

### 2.2 The 2015 ENCR-JRC data call

In 2015 a first ENCR-JRC data call was launched by the ENCR Steering Committee and JRC, as the source for data feeding the ECIS (1). Outputs at registry level reported in the ECIS web application include incidence and mortality by cancer entity, sex, age group and geographical area. Besides this information, the protocol for the 2015 ENCR-JRC data call investigated also on cancer treatment data (20). Additional information could be retrieved by answers to dedicated questions of the questionnaire accompanying the data submission.

General (all-sites) and childhood CRs contributing to the ECIS and having answered to the 2015 ENCR-JRC data call questionnaire were included in the current analysis. Site-specific registries, and regional registries overlapping with a national CR were excluded.

#### 2.2.1 The 2015 ENCR-JRC data call questionnaire

Filling the accompanying questionnaire was an essential requirement to complete the data submission. The questionnaire comprised 4 sections (Cancer case file; Population data; Mortality data and Life tables), and included in section 1 on the Cancer case file the following questions related to the registration of treatment data:

- 1.21 Do you record information about treatment in the registry?
- 1.21.1 Please, provide a description of the variables, if they are different than those in the protocol:
- 1.21.2 Please specify the sources of data on treatment:

The questionnaire was sent through the EUSurvey, the online survey management tool of the European Commission (21). Data from the submitted questionnaires were stored and analysed with Microsoft Excel.

#### 2.2.2 The 2015 ENCR-JRC data call: Treatment data reported by the CRs

The protocol of the 2015 ENCR-JRC data call included 4 variables investigating the first course of cancer therapy after diagnosis by using the following variables:

- Surgery (including any surgery to remove all or part of the cancer. Biopsy which is followed by definitive surgery was not to be included; other biopsies, where the cancer was completely excised, could be included);
- Systemic cancer therapy, including chemotherapy, targeted therapy, immunotherapy and hormone therapy;
- Radiotherapy;
- Bone marrow transplantation.

All variables were recorded with yes, no or unknown.

Data were submitted by CRs to the JRC through the ENCR-JRC Portal, checked for consistency and harmonised by JRC, using the JRC-ENCR Quality Check Software (QCS), Stata and SAS statistical software (22, 23).

A quality evaluation was performed on the four most common cancer entities (1): breast, colorectal, prostate and lung. The percentage of cancer cases with surgery was calculated by CR for each site, and compared with data previously observed in studies from CRs reporting treatment patterns.

## 3 Results

### 3.1 Literature review

#### 3.1.1 Peer-reviewed literature

A total of 2,874 articles out of 7,271 returned by the search (from year 1975 to October 2022) were included in the analysis.

The majority of papers with treatment data information came from five countries: Netherlands (632 articles - 22% of the total), Sweden (290 - 10%), United Kingdom (225 - 8%), Germany (197 - 7%) and Norway (188 - 7%) for a total of 1532 articles (53% of the total). In addition, registries operating in another 23 countries authored 912 publications (32%).

**Figure 2:**
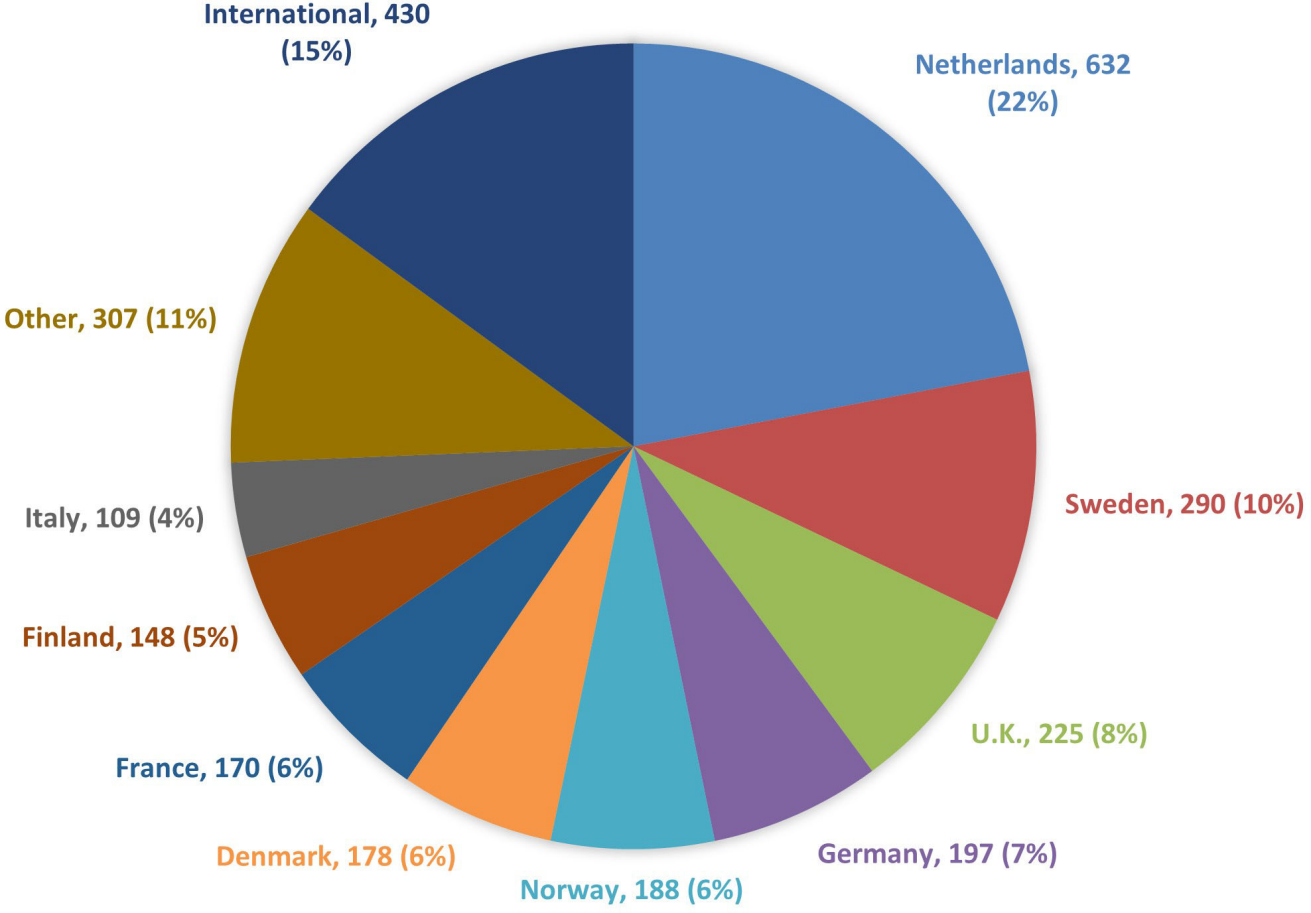
Number of articles on cancer treatment by CR country of operation, 1975-2022.

A total of 430 publications were international (15%), with data from at least two European countries (**Figure 2**). The latest ranged from large international studies such as the European Cancer Registry based study on survival and care of cancer patients (EUROCARE), the Surveillance of Rare Cancers in Europe (RARECARE/RARECAREnet), the CONCORD programme for the global surveillance of cancer survival and the European Registration of Cancer Care (EURECCA), to collaborations between CRs from as little as two different European countries.

Since many CRs started operating later than others in the period of interest (1975-2022), the analysis was also performed for the most recent period (2013-2022), for which the percentage contribution remained unchanged (**Table 1**).

**Table 1:** Number of articles on cancer treatment by CR country of operation, January 2013-October 2022.

Country	Number of articles	Percentage
Netherlands	496	27.4
Sweden	182	10.0
Germany	129	7.1
Norway	117	6.5
U.K.	109	6.0
Denmark	91	5.0
France	83	4.6
Finland	82	4.5
Italy	54	3.0
Switzerland	39	2.2
Ireland	36	2.0
Spain	27	1.5
Belgium	18	1.0
Lithuania	15	0.8
Poland	15	0.8
Czech Republic	9	0.5
Iceland	8	0.4
Portugal	8	0.4
Slovenia	7	0.4
Hungary	6	0.3
Estonia	5	0.3
Austria	4	0.2
Croatia	4	0.2
Russia	3	0.2
Bulgaria	1	0.1
Ukraine	1	0.1
International	263	14.5
Total	1812	100.0

**Figure 3:**
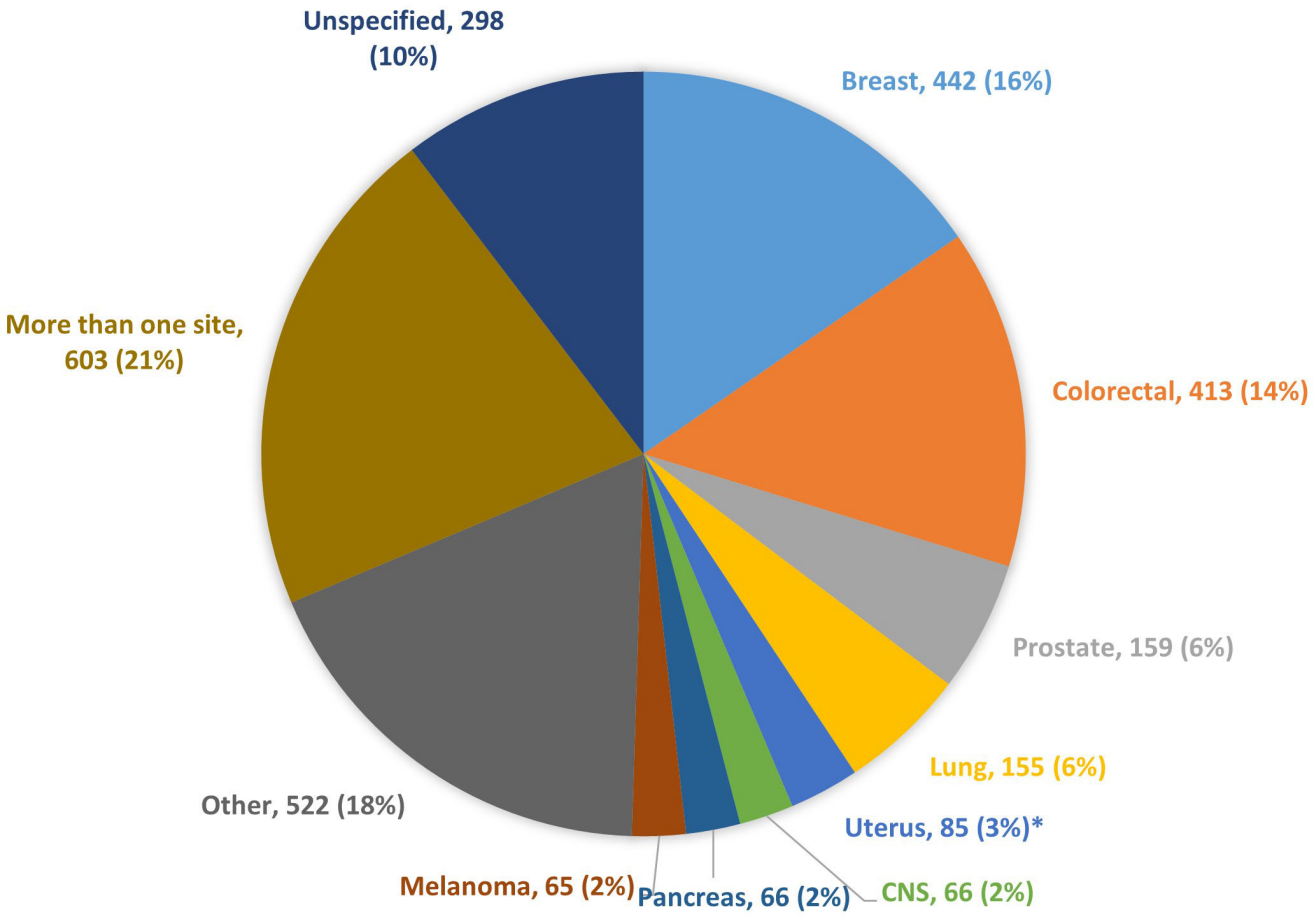
Number of articles reporting CRs information on cancer treatment, by cancer entity. * Endometrial and cervical cancers.

The highest number of articles reporting information on treatment was related to breast cancer (442 articles - 15% of the total), followed by colorectal (413 - 14%), prostate (159 - 6%) and lung cancer (155 - 5%). Additional single cancer entities were addressed in 804 articles (28%), 603 articles (21%) reported on more than one cancer entity, whereas for 298 articles (10%) the search string could not find any specific cancer entity (**Figure 3**).

**Figure 4:**
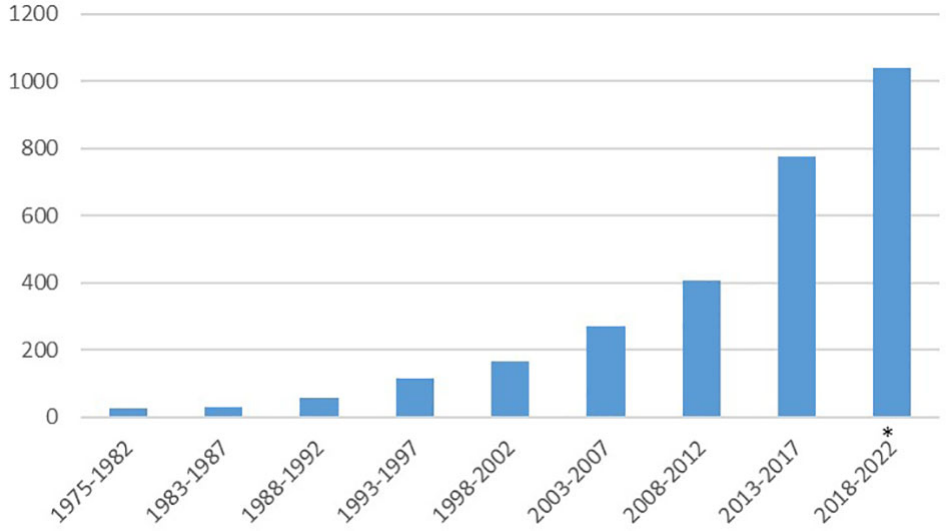
Number of articles on cancer treatment by year of publication. * Up to October 2, 2022.

Out of the total number of articles reporting cancer treatment data, 385 (13% of the total) were published between 1975 and 2002. A steep increase in the number of articles was observed in subsequent five-years periods: 269 (9%) in 2003-2007, 408 (14%) in 2008-2012, 774 (27%) in 2013-2017 and 1,038 (36%) in the latest period (January 2018-October 2, 2022) (**Figure 4**).

The selected articles were published in 599 different Journals. The 10 Journals with the highest number of papers published altogether 796 articles (28%). Epidemiology, Oncology, Surgery, specific cancer entities were the most common focus of the 599 Journals. In order to compare Journals with different periods of publication, number of articles was checked for period 2013-2022, which was already covered by the majority of Journals (**Table 2**).

**Table 2:** Number of articles on cancer treatment published in the 10 most frequent Journals, 2013-2022.

Journal (starting year publication)	Number of articles	Percentage
**Acta Oncologica (1963)**	89	4.9
**European Journal of Cancer (1965)**	77	4.3
**European Journal of Surgical Oncology (1975)**	74	4.1
**International Journal of Cancer (1966)**	57	3.2
**BMC cancer (2001)**	51	2.8
**Breast Cancer Research and Treatment (1981)**	43	2.4
**Cancer Epidemiology (1976)**	39	2.2
**British Journal of Surgery (1913)**	35	1.9
**Annals of Surgical Oncology (1994)**	33	1.8
**Colorectal disease (1999)**	33	1.8

**Figure 5:**
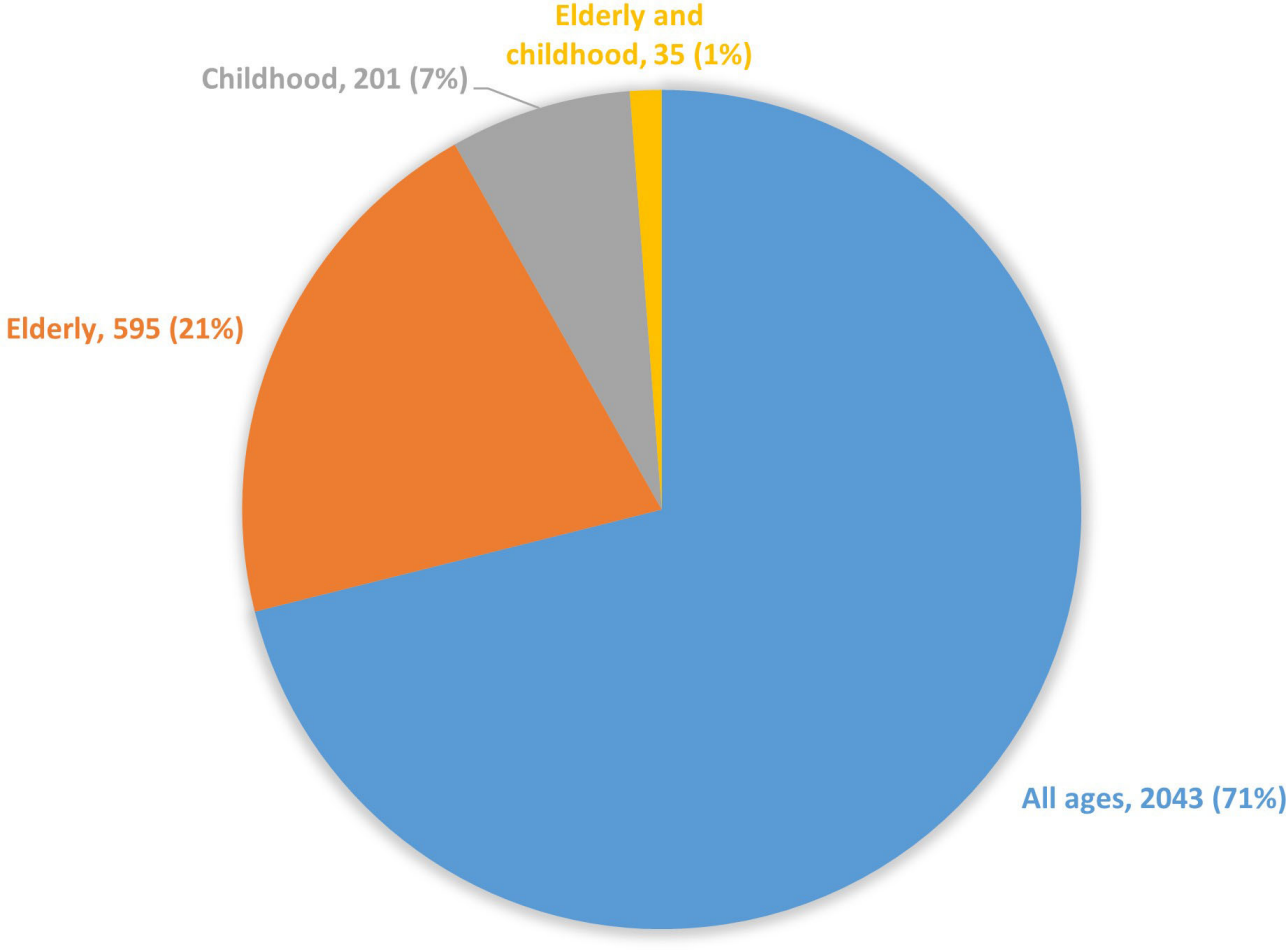
Number of articles on cancer treatment by age group, 1975-2022.

A specific search string was used to look at age groups reported in the treatment publications. The majority, 2,043 (71%), was not focused on a specific age group (in **Figure 5**, this group is shown as “All ages”). This group was in fact mainly composed of studies reporting only on adults, although this was not specifically investigated by the search string. Out of the remaining publications, 595 (21%) reported data on elderly populations with 70 years as a common threshold. Childhood populations were addressed in 201 articles (7%), and a further 35 articles (1%) reported data from both elderly and children.

#### 3.1.2 Conference proceedings

The results of the overview from the following CRs scientific meetings, having taken place between 2016 and 2019 are presented: ENCR 2016 and 2018, GRELL 2016-2019, IACR (restricted to European contributions) 2016-2019. Presentations on treatment data given by European CRs at the European Society for Medical Oncology (ESMO) Congresses 2016-2018 were also included, for a total of 213 studies (7, 24–26).

Out of 495 oral and poster presentations given at the (mainly European) GRELL and ENCR conferences in 2016-19, 132 (27%) were related to treatment.

Out of 135 CRs presentations on treatment at ENCR, IACR and ESMO (GRELL not being considered for this specific evaluation as it is only related to Latin language countries), 26 (19%) were from the Netherlands, 19 (14%) from the U.K., 11 (8%) from Belgium, 9 (7%) each from Spanish and Italian CRs. Sixteen (12%) presentations were from international studies, for the majority high-resolution ones.

Thirty-nine (18%) out of the 213 ENCR, GRELL, IACR and ESMO considered presentations were on breast cancer, 24 (11%) on colorectal cancer, 16 (8%) on lung cancer, 11 (5%) on pancreatic cancer, 10 (5%) on prostate cancer, whereas 13 (6%) took into account more than one cancer entity.

Seventy-five (35%) presentations focused on reporting treatment practice, without specific reference to guidelines, 30 (14%) on quality of care and adherence to guidelines, 26 (12%) on survival by type of treatment. Other topics addressed were the evaluation of recurrences, late effects of treatment, evaluation of new treatments at population level, new methodologies for gathering treatment data, quality of life, end-of-life care.

### 3.2 The 2015 ENCR-JRC data call

#### 3.2.1 The 2015 ENCR-JRC data questionnaire

Overall, a total of 119 general (all ages and all cancer sites) and 6 specialised childhood CRs submitted data to feed the ECIS, and responded to the 2015 data call questionnaire. Eleven additional registries submitted data to the ECIS but did not fill in the questionnaire, thus not contributing to its evaluation. Out of the 125 population-based CRs included in the analysis, 21 were national CRs while 104 were regional ones, representing a total of 30 countries.

Out of the 125 CRs, 61 (49%) replied “*Yes*” to question “*1.21 Do you record information about treatment in the registry?*”, while 64 CRs (51%) replied “*No*”.

**Figure 6:**
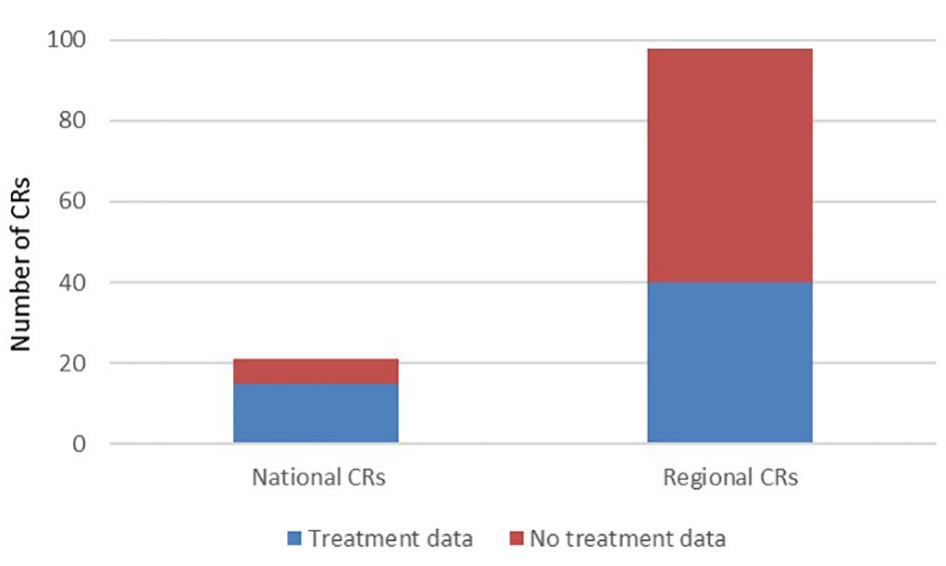
Number of general CRs reporting the recording of treatment data.

Specifically, 76% (15 out of 21) of the national general CRs reported recording treatment data, as compared to 41% (40 out of 98) of the regional CRs (**Figure 6**). In addition, all six childhood CRs reported dealing with treatment data.

Of the 61 CRs declaring to record treatment data, 59 specified the source of data on treatment. The most referenced sources were hospital discharge records (N = 23), clinical records (N = 23), both sources (N = 4) and notifications from physicians and hospitals (N = 6).

Forty-two out of the 61 CRs reporting treatment data provided additional information (question “*1.21.1 Please, provide a description of the variables, if they are different than those in the protocol:*”). Twelve registries reported that data are available but were not submitted or will only be made available for specific studies or upon request. Eleven registries commented that they had more data available than those requested in the data call, such as starting date of therapy, or additional clinical data for selected cancers and subgroups. Regarding the question about systemic treatment, CRs reported to be able to provide detailed data on the specific type of therapy, e.g. chemotherapy, hormone therapy, immunotherapy and targeted therapy. Five registries reported that they only record treatment data for specific cancers (on colorectal cancer (5 CRs), breast cancer (4 CRs), lung cancer (2 CRs), skin melanoma (1 CR) and lymphoma (1 CR)).

In addition, out of the 125 CRs included in the analysis, 98 (78%) registries reported to collect data on cancer stage. Ninety-one collected pathological or clinical TNM (Tumour/Nodes/Metastasis), 2 childhood CRs reported using specific childhood staging only, 3 CRs were only collecting summary extent of disease and 1 ‘condensed TNM’, while 1 did not provide further information on staging (27, 28).

#### 3.2.2 The 2015 ENCR-JRC data call: Information on cancer treatment

**Figure 7:**
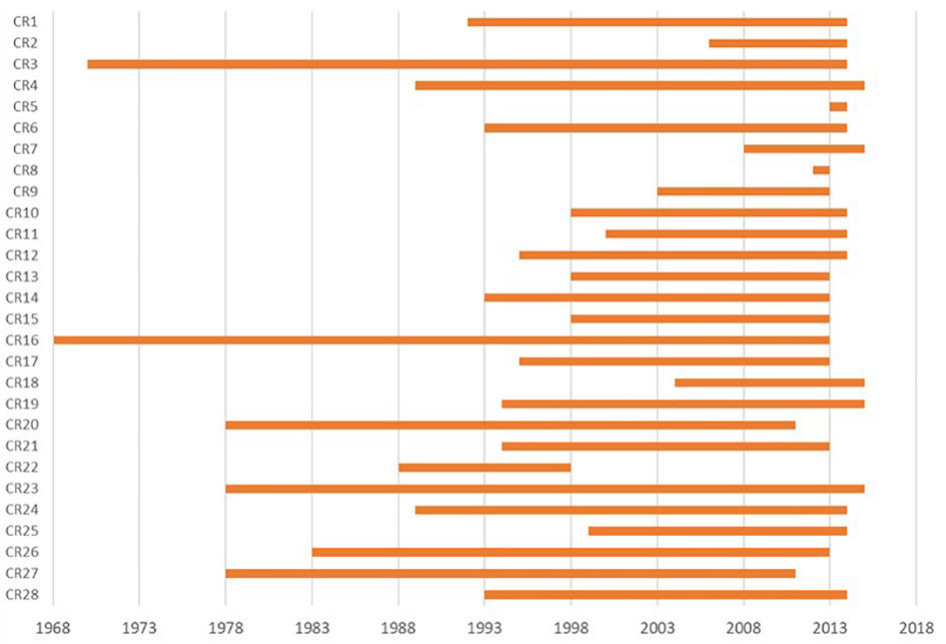
Availability of treatment information by incidence year and contributing CR in the ECIS database as of 16/10/2020.

Overall 130 registries (124 general and 6 specialised on childhood cancers) registries contributed to the ECIS database, with a total of 34,610,818 individual cancer cases as of 16/10/2020. Out of them, 30 registries (22%) - 28 general and 2 childhood CRs- provided treatment data for all or part of the period of incidence (**Figure 7**).

The number of cases provided by the 28 general CRs submitting treatment data was 12,872,032 (37% of the total). From what reported in the data call questionnaire, additional 29 general registries (22% of the total) declared to record treatment information although they did not submit it, whereas two CRs submitted treatment data but did not reply to the questionnaire.

Seven registries out of 28 provided information on all 4 treatment types as defined in the protocol for data collection (surgery, systemic therapy, radiotherapy and bone marrow transplantation), 20 registries provided data on surgery, systemic therapy and radiotherapy, and one registry provided data on surgery only. As for the cancer entities with reported data on treatment, twenty-five out of 28 CRs submitted information on surgery for lung cancer, prostate, bladder, corpus uteri, melanoma, pancreas and other cancer entities. An additional registry submitted data also on surgery for colorectal cancer; all 28 registries submitted information on surgery for breast cancer.

Out of 1,491,881 breast cancer cases submitted by the 28 general registries in the period of data availability, 82% were treated with surgery (interquartile range 79%-88%). Out of 1,464,389 colorectal cancer cases provided by the 26 registries, 71% underwent surgery (interquartile range 70%-79%). For prostate, out of 1,033,071 cases from 25 CRs, 36% received surgical treatment (interquartile range 33%-51%). As for lung cancer, out of 1,388,712 cases from 25 registries, only 19% received surgery (interquartile range 15%-22%).

## 4 Discussion

To the best of our knowledge, this is the first analysis combining data from a literature review and conference proceedings, together with data reported by the European CRs in the 2015 ENCR-JRC dataset to get the evolution over time of the status of collecting and reporting cancer treatment data in population-based CRs. Our study highlighted that population based CRs collecting treatment data either (1) do not report the data; (2) report the data but do not publish in peer-reviewed Journals; and (3) report and publish the data.

The literature review shows that there is an increase in published data on cancer treatment by population-based CRs over the years. Most articles are from CRs operating in Western and Northern European countries, notably countries with either a national CR and/or with a long history of cancer registration. In particular, in Nordic countries an extensive record linkage between different national data sources is routinely performed by CRs, which allows detailed treatment data collection and reporting (29).

In addition to the increase of publications in peer-reviewed Journals, we also notice that the vast majority of publications were in specialised Journals for clinicians, surgeons, radiation oncologists, making this data more and more relevant, at least in some European countries. This growing interaction and collaboration of clinicians and CRs could signal an increasing benefit for both the epidemiological and clinical environment.

One reason of the scarcity of articles from some European areas might be partly due to the fact that treatment data are only reported by CRs in national or regional reports, often in their respective local languages (10). In addition, many CRs gather treatment data only for *ad hoc* projects, such as the EUROCARE, RARECAREnet and CONCORD high resolution studies or the EURECCA studies (30–32).

Moreover, limited resources in some European countries and regions could play a role in the difference in reporting and using treatment data among European CRs as well as less developed or absent national linkage/database structures (33).

As for the specialised childhood registries, given the much lower number of incident cases (e.g. 16.000 estimated in 2020, compared to 4 million for adults), registration and use of treatment data is more widespread than for the general CRs. The literature review showed that 8% of articles report data on treatment for the paediatric age groups, whereas childhood cancers represent only 0.4% of total incidence.

The literature review also revealed that a consistent (21%) proportion of publications from CRs is reporting data on elderly patients. Cancer cases in people aged 70 years or above represented more than 47% of the total EU-27 estimated incidence for 2020 (1), making this group underrepresented in the literature. This underreporting might be also related to the fact that in some countries treatment of elderly cancer patients is administered in settings such as hospices or to homecare services that are not reported in health care records and are not regularly accessible to the CRs. It is anyhow important to have identified such publications, since elderly people are usually even more underrepresented in clinical trials. CRs can indeed offer an added value and complementarity with clinical studies, helping exploring treatment strategies in the elderly (32).

The literature review and conference proceedings revealed that treatment data are most often collected for breast cancer, the most frequent cancer in women in Europe. Treatment data are often collected for colorectal, prostate and lung cancers, which are also among the most common. Data in the literature review are also consistent with the results derived from the dataset of the 2015 ENCR-JRC data call, where all registries reported treatment data for breast cancer, while most registries reported treatment data for colorectal, prostate and lung cancer.

Prior to the 2015 ENCR-JRC data collection, two other projects (EUROCHIP-3 and EUROCOURSE) provided information on the availability and use of treatment data in European CRs. The EUROCHIP-3 survey was carried out during 2010 and presented an overview on the availability of three main indicators in European population-based CRs: stage at diagnosis, cancer treatment delay and compliance with cancer guidelines. Information on treatment data was available in 30% of the 86 responding registries (4). The second project, EUROCOURSE (2010-2012), reported that 43% of the 106 responding registries gathered information on first treatment (5). The 2015 ENCR-JRC questionnaire reported that 49% of the 125 responding registries collect cancer treatment data. This proportion is higher (52%) if the site-specific and regional CRs overlapping with national ones is considered. A steady increase in the percentage of CRs collecting treatment data is therefore observed over the three data collection periods. A possible reason for this is the rising number of European countries and regions using electronic health records, which can be used for research purposes (33).

Evidence from the results of the ENCR-JRC 2015 data collection suggests that national registries are collecting cancer treatment data more frequently as compared to regional registries. This is consistent with the fact that usually national CRs have more resources available, either technical, financial and/or human. It was also observed that while 61 CRs reported in the questionnaire to collect treatment data, only 28 CRs actually submitted such data in the 2015 ENCR-JRC data call. Twelve registries indeed mentioned in the questionnaire that they collect treatment data but did not submit them, mainly motivating this with data incompleteness. This underreporting behaviour calls for increased awareness among the CRs on the importance in reporting treatment information.

While half of the general CRs responding to the 2015 call reported to collect treatment data, four out of five reported to collect data on cancer stage. According to the data call questionnaire, the six childhood CRs included in our analysis report to collect data on both cancer stage and treatment. Overall, more CRs are reporting data on cancer stage as compared to treatment. This finding is consistent with earlier research reporting that 61% of responding CRs collected data on cancer stage, while 43% reported cancer treatment data (5). The higher number of CRs recording stage compared to those reporting stage and treatment, is likely related to the fact that stage information has been standardised with the introduction of the TNM classification system already in the 1940s. There are also extensive training materials and activities on TNM coding, which explain its diffusion among European CRs. Such standardisation has not yet been performed thoroughly for the coding and registration of treatment data. Consensus guidelines for staging childhood cancers (the Toronto Paediatric Cancer Stage Guidelines) have been developed and endorsed for use by CRs. The international project ‘BENCHISTA’ involves most of the European CRs and is a good example of how to standardise the collected information for clinical variables like treatment and stage (34, 35).

The analysis of treatment data provided by the 28 general CRs contributing to the ECIS database revealed that for the main solid tumours the proportion of cancer patients treated with surgery was: 82% for breast cancer, 71% for colorectal, 36% for prostate and 19% for lung cancer. These results are consistent with previously published evidence on the impact of surgical treatment in Europe and in the USA (36–40).

In the recent years exploratory analyses on treatment in Europe by stage, age group, sex, period of incidence and geographical area from the ECIS database were carried out, addressing specifically breast, colorectal, prostate, endometrium and glioblastoma. Such analyses investigated to what extent some selected clinical and treatment patterns by age group, stage and period could be monitored using the 2015 ENCR-JRC dataset (40–45).

A limitation in the literature review could be the focus on the proceedings from scientific conferences of only four international societies, and the lack of other grey literature such as reports on CRs websites, or in languages other than English.

A further limitation was given by the use of search strings: although checks were performed on the results, the search method reduced the level of detail of the results from the review. Lastly, only titles and abstracts were reviewed, thus losing potential information from the full articles’ text.

Regarding the 2015 ENCR-JRC dataset, one limitation consisted in the impossibility to distinguish between chemotherapy and other types of systemic therapy. This issue has been addressed in the new 2022 ECIS call for data protocol, where information on timing (neoadjuvant or adjuvant therapy), crucial for monitoring clinical care, has been added; systemic therapy information has been split in different variables (chemotherapy, targeted therapy, immunotherapy, hormone therapy, other/unspecified), and surgery has been detailed between local surgery and operative surgery (46).

In order to use treatment information and to ensure its quality and comparability at European level, a more harmonised collection of these variables among European population-based CRs is required. In fact, the availability of comparable information on treatment (and stage at diagnosis) is crucial to improve the interpretation of cancer outcome disparities between populations, therefore bringing valuable real life information for patients, clinicians, policymakers and other stakeholders.

## 5 Conclusion and way forward

Treatment data are increasingly being reported by CRs, though further improvements are needed to ensure complete and harmonised coverage of such important information. Sufficient technical, financial and human resources are needed to collect treatment data in a harmonised way, while clear guidelines for treatment data collection need to be developed.

To address these challenges, the ENCR Working Group on Treatment Data Harmonisation was set up in June 2021, with the aim of bringing together European experts in cancer registration, epidemiology and from the clinical field to discuss and draft guidelines for improved data collection and harmonisation of treatment data among European population-based CRs. This ongoing activity will be a key step to provide cross-comparisons between European regions and countries, contributing to design actions to ensure better integrated and comprehensive cancer care and addressing unequal access to optimal care, namely the ultimate goal of the European Commission’s Europe’s Beating Cancer Plan (47).

## 6 The ENCR working group on treatment data harmonisation

Francesco Giusti (European Commission/Belgian Cancer Registry), Carmen Martos (European Commission/FISABIO, Valencia, Spain), Otto Visser (Netherlands Comprehensive Cancer Organisation - IKNL), Lisbeth Van Eycken (Belgian Cancer Registry), Claudia Allemani (London School of Hygiene and Tropical Medicine), Volker Arndt (German Cancer Research Centre - DKFZ), Riccardo Audisio (Sahlgrenska University Hospital, Göteborg, Sweden), Manola Bettio (European Commission), Roberta De Angelis (Istituto Superiore di Sanità, Rome, Italy), Henna Degerlund (Finnish Cancer Registry), Silvia Francisci (National Health Institute, Rome, Italy), Gemma Gatta (Fondazione IRCCS Istituto Nazionale dei Tumori, Milan, Italy), Anna Gavin (Northern Ireland Cancer Registry), Tom Børge Johannesen (Cancer Registry of Norway), Yolande Lievens (Ghent University Hospital and Ghent University, Ghent, Belgium), Margit Mägi (Estonian Cancer Registry), Rafael Marcos-Gragera (Epidemiology Unit and Girona Cancer Registry, Catalan Institute of Oncology, Girona, Spain), Eva Morris (Nuffield Department of Population Health, Big Data Institute, University of Oxford, UK), Regina Nanieva (National Institute for Cancer Epidemiology and Registration - NICER, Switzerland), Raquel Negrão Carvalho (European Commission), Laura Pareja Fernandez (Department of Health of Catalonia, Hospitalet del Llobregat, Barcelona, Spain), Francesco Pignatti (European Medicines Agency), Josepa Ribes (Department of Health of Catalonia, Hospitalet del Llobregat, Barcelona, Spain), Silvia Rossi (Istituto Superiore di Sanità, Rome, Italy), Arantza Sanvisens (Epidemiology Unit and Girona Cancer Registry, Catalan Institute of Oncology, Girona, Spain), Annalisa Trama (Fondazione IRCCS Istituto Nazionale dei Tumori, Milan, Italy), Maciej Trojanowski (Greater Poland Cancer Registry), Ulrich Wagner (National Institute for Cancer Epidemiology and Registration – NICER, Switzerland), Paul Walsh (National Cancer Registry Ireland), Vesna Zadnik (Epidemiology and Cancer Registry, Institute of Oncology Ljubljana, Slovenia).

## Data availability statement

The datasets presented in this article are not readily available because aggregated data has been used; individual patient data is not available. Requests to access the datasets should be directed to francescogiusti@hotmail.com.

## Author contributions

The first draft of the manuscript was written by FG, CM and LV. All authors contributed to the article and approved the submitted version.
